# Association of risk factors with mental illness in a rural community: insights from machine learning models

**DOI:** 10.1192/bjo.2025.47

**Published:** 2025-05-12

**Authors:** Firoj Al-Mamun, Mohammed A. Mamun, Md Emran Hasan, Moneerah Mohammad ALmerab, Johurul Islam, Mohammad Muhit

**Affiliations:** CHINTA Research Bangladesh, Savar, Dhaka, Bangladesh; Department of Public Health & Informatics, Jahangirnagar University, Savar, Dhaka, Bangladesh; Department of Public Health, University of South Asia, Dhaka, Bangladesh; School of Computer Science and Engineering, Nanjing University of Science and Technology, Nanjing, China; Department of Psychology, College of Education and Human Development, Princess Nourah Bint Abdulrahman University, Riyadh, Saudi Arabia; CSF Global, Banani, Dhaka, Bangladesh

**Keywords:** Mental health, machine learning, rural community, depression, anxiety

## Abstract

**Background:**

Mental health conditions, particularly depression and anxiety, are highly prevalent and impose substantial health burdens globally. Despite advancements in machine learning, there is limited application of these methods in predicting common mental illnesses within community populations in low-resource settings.

**Aims:**

This study aims to examine the prevalence and associated risk factors of common mental illnesses collectively (depression and anxiety) in a rural Bangladeshi community using machine learning models.

**Method:**

This cross-sectional study surveyed 490 adults aged 18–59 in a rural Bangladeshi community. Depression and anxiety were assessed using the Patient Health Questionnaire (PHQ-2) and Generalised Anxiety Disorder (GAD-2) scales. Machine learning models, including Categorical Boosting, the support vector machine, the random forest and XGBoost (eXtreme Gradient Boosting), were trained on 80% of the data-set and tested on 20% to evaluate predictive accuracy, precision, F1 score, log-loss and area under the receiver operating characteristic curve (AUC-ROC).

**Results:**

Some 20.4% of participants experienced at least one common mental illness. Feature importance analysis identified house type, age group and educational status as the most significant predictors. SHAP (Shapley Additive exPlanations) values highlighted their influence on model outputs, and the XGBoost gain metric confirmed the importance of marital status and house type, with gains of 0.76 and 0.73, respectively. XGBoost delivered the best performance, achieving an F1 score of 71.01%, precision of 71.58%, accuracy of 71.15% and the lowest log-loss value of 0.56. The random forest had an accuracy of 78.21% and an AUC-ROC of 0.90.

**Conclusions:**

The findings of this study suggest targeted interventions addressing housing and social determinants could improve mental health outcomes in similar rural settings. Further studies should consider longitudinal data to explore causal relationships.

Good mental health is crucial for individuals to effectively cope with life’s challenges and make meaningful contributions to their communities. However, exposure to adverse circumstances, such as poverty, violence, disability and inequality, increases the likelihood of developing mental health conditions. In recent years, there has been a 13% increase in reported mental health issues worldwide.^1^ The World Health Organization recognises depressive and anxiety disorders, affecting 12.5% of the global population. In 2019, over 300 million had anxiety disorders, and 280 million had depressive disorders.^1^ Global costs of these disorders are US$1 trillion annually,^1^ and less than 2% of government health expenditure is allocated to mental healthcare.^2^ These mental health conditions can profoundly affect various life aspects, including work performance, academic achievement, relationships and community involvement.

Recent advancements in machine learning have significantly affected mental health diagnostics and treatment. Shatte et al^3^ highlight machine learning’s broad application in detecting and diagnosing mental health conditions, while Iyortsuun et al^4^ emphasise its role in predicting treatment outcomes for disorders such as depression and anxiety. For instance, Cho et al^5^ studied depression in the Korean community using National Health and Nutrition Examination Survey (NHANES) data, while Zhang et al^6^ focused on depression in middle-aged and elderly populations in the USA. In addition, Oh et al^7^ examined depression across both data-sets. Dipnall et al^8^ used machine learning to explore the association between medical symptoms and depression, highlighting the importance of bowel-related symptoms. Kim et al^9^ investigated machine learning models for predicting depression among Korean employees, highlighting the relevance of job-related and psychosocial factors in predicting depression, suggesting that machine learning can be used to develop intelligent systems for workplace mental health monitoring. In the field of anxiety research, Tabares et al^10^ conducted a study on young people in Colombia and found that socio-familial factors such as parental education level, alcohol consumption and social security affiliation were the strongest predictors. Another study among adolescents identified key predictors such as psychometric features, including neuroticism, hopelessness and emotional symptoms,^11^ and loneliness and self-esteem as significant predictors were reported in another study among elderly Korean populations.^12^

Despite advancements in machine learning, there remains a significant gap in its application to predict and understand mental health conditions within community populations. A review by Shatte et al^3^ highlighted that most existing studies predominantly focus on depression, with limited research addressing anxiety. Moreover, no studies have examined the presence of at least one disorder, such as depression or anxiety, within community settings in Bangladesh. Thus, this study aims to fill this gap by using machine learning techniques to identify models that best predict mental illness in a rural community. Besides, the study evaluates factors influencing mental illness using both machine learning and traditional statistical approaches. By focusing on community contexts, this research provides new insights into the prevalence and determinants of these common mental health conditions. The findings aim to inform targeted interventions and advance the understanding and management of mental health in diverse and resource-limited populations.

## Method

### Study design and participants

The current study used a cross-sectional design and a household survey to assess the prevalence of common mental health disorders among rural communities in Bangladesh, from the project entitled *BD ComMen Study*. Data were collected in May 2022 by a trained research team through face-to-face interviews. Approximately 585 responses were collected, with 490 participants retained for final analysis after removing incomplete questionnaires. The inclusion criteria were participants aged between 18 and 59 years old. People who were unwilling to participate were excluded.

### Sampling frame

Bera Upazila of the Pabna district was selected for this study because of logistical consideration and the feasibility of conducting the study within this area. The demographic diversity of Bera Upazila makes it a reasonable proxy for other rural areas in Bangladesh. Subsequently, a random sampling method was employed. One union was randomly selected from the nine unions in Bera Upazila. After that two wards were randomly selected from the wards within the chosen union using the lottery method. This random selection process ensured that each ward had an equal chance of being selected, thereby eliminating the representativeness of the sample. These wards were considered clusters for recruiting adults aged 18–59 years.

### Sample size calculation

The sample size was calculated using Cochran’s formula for prevalence studies, which is widely recognised and utilised in epidemiological research. The formula is as follows:

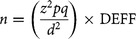

where *n* is the sample size, *z* = 1.96 is the 95% confidence interval, *p* is prevalence, 16.5%;^13^ *q* = (1 –*p*); *d* = 5% is the margin of error and a there is a design effect of 1 arising from the clustering of participants within wards since the clusters were within a single union. This formula yielded an estimated sample size of 212. After adjusting for sampling errors with a 10% non-response rate, the estimated sample size was 232 for each cluster. The present study comprised 490 participants, which showed an adequate sample size.

### Measures

#### Sociodemographic factors

Sociodemographic information, including age, gender, marital status, level of education, monthly family income, occupation, debt, type of housing, presence of any chronic conditions, family history of mental health and family history of suicide, was gathered. Monthly family income was categorised as low income (less than 15 000 Bangladeshi Taka (BDT)), middle income (15 000 to 30 000 BDT) and high income (more than 30 000 BDT), and converted to the US$ equivalent. House type was categorised based on ownership, such as own house and house provided by the government based on financial eligibility. This classification aimed to capture the economic contexts of housing but may not fully account for variations in living arrangements across age groups, such as younger individuals sharing housing with their parents. Besides, we collected data on personal COVID-19 infection, as well as infection or death of friends or family members, with responses in a yes/no format.

#### Mental health problems

In the study, standardised scales were used to assess depression and anxiety: the Patient Health Questionnaire (PHQ-2)^14^ and the Generalised Anxiety Disorder (GAD-2) scale.^15^ Both scales utilise a 4-point Likert scale ranging from 0 (not at all) to 3 (nearly every day), resulting in a total score range of 0–6. A cut-off score of ≥3 was used to identify probable cases of depression or anxiety. The reported Cronbach’s alpha for each scale was 0.76. The study’s outcome variable was ‘any mental illness’, indicating the presence of at least one of the following conditions: depression or anxiety.

### Machine learning models

#### *k*-nearest neighbours

The *k*-nearest neighbours (KNN) algorithm defers computation until after classification and uses locally estimated functions to solve regression and classification problems. It identifies the *k* training samples in the feature space that are most similar to the item being classified and assigns the class membership based on a majority vote from its KNN, with *k* being a small positive number. When *k* is 1, the item is assigned to the class of its closest neighbour.^16^

#### Random forest

The random forest method is a popular ensemble learning technique for regression and classification problems that constructs multiple decision trees during training and outputs predictions by averaging (for regression) or majority voting (for classification) across all trees. This approach reduces overfitting and enhances generalisation by aggregating the results of individual trees. Random forest models improve prediction accuracy by using different portions of the training data. As a result, this diverse forest of trees produces a more accurate model.^17^

#### Gradient boosting algorithms: gradient boosting machines and eXtreme Gradient Boosting

XGBoost (eXtreme Gradient Boosting) is an optimised implementation of the gradient boosting machine (GBM), designed for improved speed and accuracy. Both XGBoost and GBMs are gradient boosting algorithms that build models sequentially, with each new model correcting errors made by the previous ones. XGBoost is an advancement in ensemble machine learning techniques, outperforming traditional methods. It enables the sequential construction of decision trees, reducing errors by learning from prior mistakes.^18^ Predictive modelling has advanced with XGBoost, surpassing the accuracy of its predecessors because of its systematic approach to speed and performance enhancement and its skilful handling of large-scale data.^19^ A powerful machine learning technique known as the GBM sequentially adds weak learners, typically decision trees, to build a strong learner. This method uses gradient descent to minimise the loss function and is effective for a range of predictive applications. Careful hyperparameter modification is needed to prevent overfitting, but GBMs are widely used in various industries because of their ability to handle complex, nonlinear data.^19^

#### Categorical Boosting

Categorical Boosting (CatBoost) is a contemporary machine learning approach developed by Yandex. CatBoost is a gradient boosting algorithm specifically designed to handle categorical data efficiently. It works well with classified data, particularly data-sets with a high concentration of categorical variables. CatBoost minimises typical issues with categorical data without requiring extensive preprocessing by combining one-hot encoding with an advanced algorithmic technique to reduce overfitting and improve prediction accuracy. Unlike traditional methods that rely on one-hot encoding, CatBoost uses a technique called ordered boosting, which reduces target leakage and improves model performance. This approach is known for its scalability and efficacy, making it a valuable tool for various applications, such as predictive modelling and recommendation systems.^20^

#### Support vector machine

The support vector machine (SVM) is a robust supervised learning technique that is frequently applied to classification and regression tasks. It maximally divides the different class memberships in a data-set by finding the optimal hyperplane. The SVM uses the data points in the support vectors that are closest to the decision border to increase classification accuracy. The SVM employs the kernel trick to transform nonlinear data into a higher-dimensional space, where linear separation becomes possible. This allows the SVM to effectively handle complex, nonlinear decision boundaries.^21^

### Statistical analysis

Data analysis was conducted using IBM SPSS software version 25 for Windows (IBM Corp., Armonk, NY, USA). Descriptive statistics (i.e. frequency and percentages) and inferential statistics (chi-square, Fisher exact test and logistic regression) were carried out to analyse the data. The association between the study variables and the outcomes was estimated using either the chi-square test or Fisher’s exact test. Fisher’s exact test was used when more than 20% of cells have <5 expected frequencies. Binary logistic regression analysis was conducted to examine the associated factors with the outcome variables and results were reported as odds ratios. A *p*-value of <0.05 was set as statistical significance with a 95% confidence interval.

#### Machine learning analysis

In this study, the contributing factors to depression and anxiety in a rural community were examined using machine learning approaches. Python, the primary programming language, was used in conjunction with Google Colab to analyse the data. The data-set was divided into two categories: 80% for training and 20% for testing because of its relatively small size. Machine learning analysis uses SMOTE (Synthetic Minority Oversampling Technique) to address class imbalance. By creating artificial samples for the minority class, this technique balances the data-set and enhances model evaluation and performance. Through implementation, the predictive power of a number of machine learning models, including XGBoost, CatBoost, KNN, the random forest, the SVM and the GBM, was evaluated. The precision, accuracy, F1 score, log-loss metrics and area under the receiver operating characteristic curve, or AUC-ROC, were among the metrics used to assess each model’s performance. To ensure robust evaluation of model performance and prevent data leakage, *k*-fold cross-validation was carried out with *k* = 5. The results presented in this manuscript are based on the average performance across all folds. This approach allows the models to be tested on multiple subsets of the data, ensuring a reliable and generalisable estimate of their effectiveness. By systematically rotating the training and validation sets, this method minimises bias and provides a more accurate assessment of model performance.

The models in this study were chosen based on their strengths in handling structured data, robustness and ability to capture complex patterns. The random forest was chosen because of its interpretability and ensemble nature. The GBM and XGBoost were added because of their scalability and sequential error correction, with XGBoost providing extra optimisations such as parallel processing. CatBoost was selected because of its effective handling of categorical data through its ordered boosting approach. The kernel trick was used to determine the effectiveness of the SVM in high-dimensional, nonlinear spaces. Lastly, KNN was assessed for its ease of use and capacity to identify local data trends. Together, these models provide a comprehensive evaluation of traditional and state-of-the-art approaches.

#### Feature selection

**Fig. 1:**
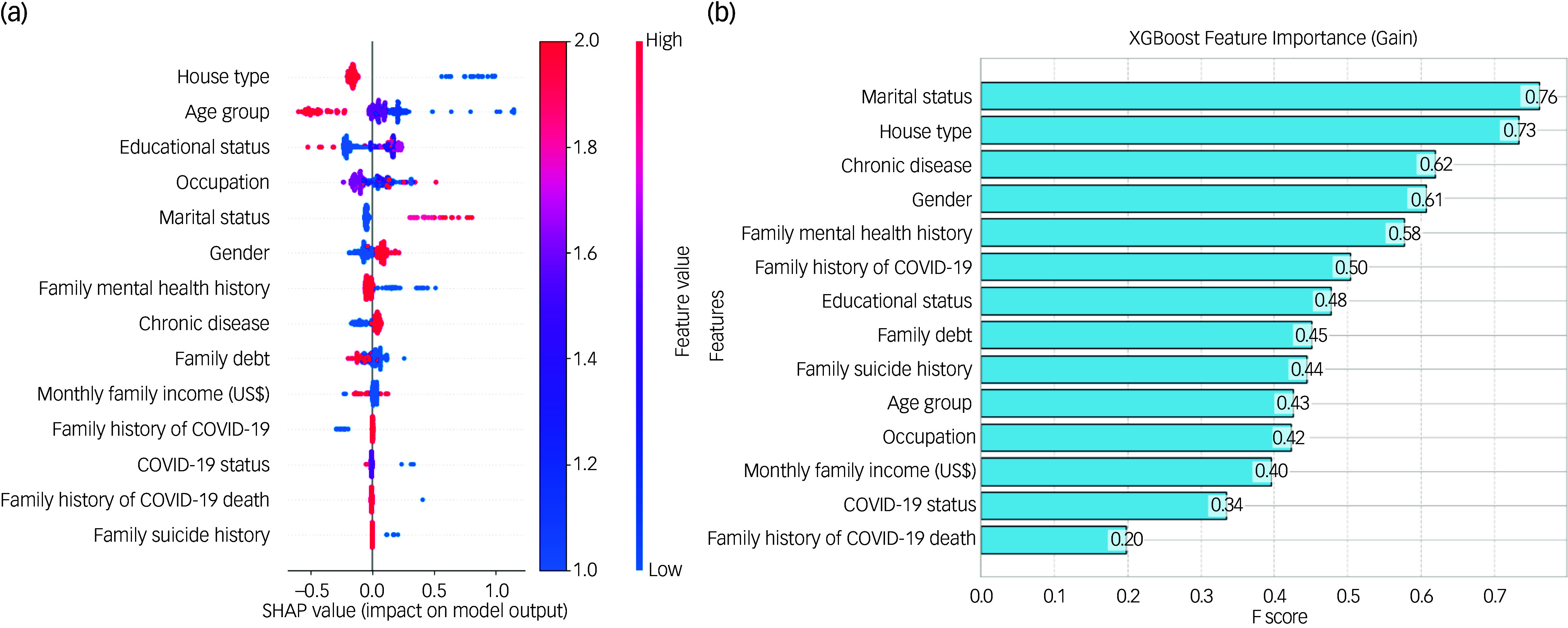
The impact of features on the model by Categorical Boosting SHAP (Shapley Additive exPlanations) value, and eXtreme Gradient Boosting (XGBoost) feature importance based on gain.

Figure 1(a) shows the SHAP (Shapley Additive exPlanations) values produced by the CatBoost model, which show how different characteristics affect the model’s output. Every feature is prioritised based on how important it is, and each feature’s contribution to the prediction is indicated by its SHAP value. While some characteristics, including family history of COVID-19 death and family suicide history, had less of an impact, others such as house type, age group and educational status have greater SHAP values, indicating significant influence. These factors have considerable effects. This analysis improves the interpretation of the model’s predictions, which helps determine the relative significance of each feature.

The XGBoost model’s gain metric is used to illustrate the feature relevance in Fig. 1(b). A feature’s gain is shown by its F score, and the bar chart ranks features based on how much of a contribution they make to the prediction performance of the model. At gains of 0.76 and 0.73, respectively, marital status and house type emerge as the most significant features. With improvements of 0.62 and 0.61, chronic disease and gender, respectively, are two other noteworthy characteristics. These results underscore these variables’ significance in the predictive analysis and show how crucial a part they play in the model’s judgements. The relative significance of each attribute in improving the accuracy and dependability of the model is made easier to understand with the use of this representation.

### Ethics statement

The authors assert that all procedures contributing to this work comply with the ethical standards of the relevant national and institutional committees on human experimentation and with the Helsinki Declaration of 1975, as revised in 2013. All procedures involving human participants were approved by ethics committee at the University of South Asia (Ref: USA-2022(1)). Before commencing the interviews, informed consent – either verbal or written – was obtained from all participants. Verbal consent was witnessed and/or formally recorded. No monetary or non-monetary incentives were provided. Participants were assured that their records would remain confidential.

## Results

### Characteristics of the study participants

Of the 490 participants in the study, 53.5% were female. The mean age of participants was 36.97 ± 10.56 years, with the majority falling in the 31–33 years age group (42%). Some 88.8% were married, 42.9% had no formal education, 47.8% were housewives and 84.7% had a monthly family income of up to US$160.68. Some 91.2% lived in their own house, 64.3% reported having family debt, 26.3% had a chronic disease, 13.3% had a family mental illness history and 2.9% had a family history of suicide. Besides, 1.4% had COVID-19 infection, 4.9% had a family history of COVID-19 infection and 1.2% reported the death of family members or friends caused by COVID-19 (Table 1). The overall prevalence of depression was 11.2%, and that of anxiety was 12.2%. About 20.4% of the participants had reported suffering from any mental illness.

**Table 1:** Association and factors associated with any mental illness and the study variables

Study variables	Total sample, (*n*, %)	Any mental illness			Logistic regression	
		Yes, (*n*, %)	*χ* ^2^ value	*p*-value	Adjusted odds ratio (95% CI)	*p*-value
Age group						
18–30 years	143, 29.2	27, 18.9	0.229	0.892	0.750 (0.342–1.644)	0.669
31–44 years	206, 42	42, 20.4			1.014 (0.544–1.893)	
45–59 years	118, 24.1	25, 21.2			Ref.	
Gender						
Male	228, 46.5	44, 19.3	0.323	0.570	0.253 (0.079–0.811)	**0.021**
Female	262, 53.5	56, 21.4			Ref.	
Marital status						
Married	435, 88.8	81, 18.6	10.737	**0.005**	0.306 (0.109–0.860)	0.048
Unmarried	31, 6.3	9, 29.0			0.594 (0.114–3.095)	
Divorcee/separated/widow	22, 4.5	10, 45.5			Ref.	
Educational status						
No formal education	210, 42.9	40, 19.0	2.543	0.637	1.840 (0.174–19.502)	0.304
Primary level	174, 35.5	38, 21.8			2.662 (0.256–27.687)	
Secondary level	60, 12.2	15, 25.0			3.720 (0.361–38.364)	
Higher secondary level	31, 6.3	6, 19.4			1.331 (0.104–17.046)	
Bachelor and above	13, 2.7	1, 7.7			Ref.	
Occupation						
Farmer	73, 14.9	10, 13.7	10.544	0.104	0.632 (0.088–4.534)	0.284
Day labour	66, 13.5	19, 28.8			1.123 (0.164–7.691)	
Businessman	67, 13.7	14, 20.9			1.021 (0.146–7.129)	
Homemaker	234, 47.8	45, 19.2			0.255 (0.032–2.036)	
Employed	12, 2.4	–			–	
Student	27, 5.5	8, 29.6			1.141 (0.115–11.336)	
Others	9, 1.8	3, 33.3			Ref.	
Monthly family income						
Up to US$160.68	415, 84.7	88, 21.2	0.729	0.695	1.070 (0.198–5.776)	0.665
US$160.68–321.37	60, 12.2	10, 16.7			0.685 (0.105–4.459)	
More than US$321.37	10, 2	2, 20.0			Ref.	
House type						
Government-provided	36, 7.3	14, 38.9	8.320	**0.004**	3.112 (1.384–6.998)	**0.006**
Own	447, 91.2	84, 18.8			Ref.	
Family debt						
Yes	315, 64.3	67, 21.3	0.330	0.566	0.965 (0.558–1.667)	0.897
No	173, 35.3	33, 19.1			Ref.	
Chronic disease						
Yes	129, 26.3	33, 25.6	2.740	0.098	1.007 (0.544–1.863)	0.983
No	358, 73.1	67, 18.7			Ref.	
Family mental health history						
Yes	65, 13.3	19, 29.2	3.515	0.061	1.626 (0.754–3.507)	0.215
No	423, 86.3	81, 19.1			Ref.	
Family suicide history						
Yes	14, 2.9	3, 21.4	0.008	1.000^a^	0.857 (0.184–3.992)	0.844
No	475, 96.9	97, 20.4			Ref.	
COVID-19 status						
Yes	7, 1.4	2, 28.6	0.395	0.821	1.703 (0.137–21.096)	0.917
No	469, 95.7	95, 20.3			1.293 (0.244–6.859)	
Did not know or did not test	12, 2.4	2, 16.7			Ref.	
Family history of COVID-19						
Yes	24, 4.9	3, 12.5	0.946	0.440^a^	0.311 (0.062–1.562)	0.156
No	464, 94.7	96, 20.7			Ref.	
Family history of COVID-19 death						
Yes	6, 1.2	1, 16.7	0.051	1.000^a^	0.805 (0.074–8.710)	0.858
No	480, 98	98, 20.4			Ref.	

a. Fisher’s exact test.

Bold values represent significant results.

### Associations of the study variables with any mental illness

Table 1 shows associations between sociodemographic and other variables and the presence of mental illness. Marital status showed a significant relationship, where divorced, separated or widowed individuals had a substantially higher prevalence of mental illness (45.5%) compared to married (18.6%) and unmarried individuals (29.0%) (*χ* ^2^ = 10.737, *p* = 0.005). Besides, house type was strongly associated with the presence of any mental health problem, with those residing in government-provided housing showing a significantly higher prevalence (52.8%) compared to those living in their own homes (18.8%; *χ* ^2^ = 8.320, *p* = 0.004).

### Factors associated with any mental illness

Table 1 presents the factors associated with any disorder, highlighting significant findings from both unadjusted and adjusted models. In the adjusted analysis, gender was a significant predictor, where males were found to have significantly lower odds of developing mental illness compared to females (adjusted odds ratio (AOR) 0.253, 95% CI: 0.079–0.811, *p* = 0.021). House type also remained a significant factor in the adjusted model, with individuals living in government-provided housing being over five times more likely to have a psychiatric disorder compared to those living in their own homes (AOR 3.112, 95% CI: 1.384–6.998, *p* = 0.006).

### Evaluation of machine learning model performances

Table 2 displays the machine learning models’ predicted performance indicators for mental illness. The capacity of each model to forecast any disorder was demonstrated after a comprehensive analysis that included accuracy precision, F1 score and log-loss measures. Notably, all of the algorithms yielded reasonable performance metrics. The random forest scored the highest accuracy of 78.21%, while the KNN and SVM models scored the lowest accuracy of 67.95%, respectively. Besides, XGBoost achieved the same accuracy score of 71.15%. In a similar precision score, KNN scored the lowest 67.97%, while the random forest scored the highest 78.68%, and XGBoost achieved 71.58%. In terms of F1 score, the random forest has the highest score of 78.12%, while the SVM has the lowest score of 67.52%, and XGBoost also achieved a good score of 71.01%. Furthermore, all algorithms showed acceptable logarithmic loss rates in every scenario, indicating incredibly precise and secure model predictions. Notably, XGBoost had the highest forecast accuracy for any disorder and the lowest log-loss of 0.56. Despite, the GBM log-loss of 0.60, CatBoost log-loss of 0.62 and SVM log-loss of 0.63, they also achieved lower scores, while KNN had the highest log-loss of 1.18. In every category throughout the study, the XGBoost model outperformed the other models, demonstrating its greater prediction power. Its higher performance over other models was probably influenced by its capacity to manage categorical variables efficiently and prevent overfitting.

**Table 2:** Evaluation of machine learning model performances

Model	Any disorder			
	Accuracy	Precision	F1 score	Log-loss
KNN	67.95	67.97	67.93	1.18
Random forest	78.21	78.68	78.12	0.69
XGBoost	71.15	71.58	71.01	0.56
CatBoost	69.87	70.28	69.72	0.62
GBM	69.87	70.14	69.77	0.60
SVM	67.95	68.96	67.52	0.63

KNN, k-nearest neighbour; XGBoost, eXtreme GradientBoosting; CatBoost, Categorical Boosting; GBM, gradient boosting machine; SVM, support vector machine.

**Fig. 2:**
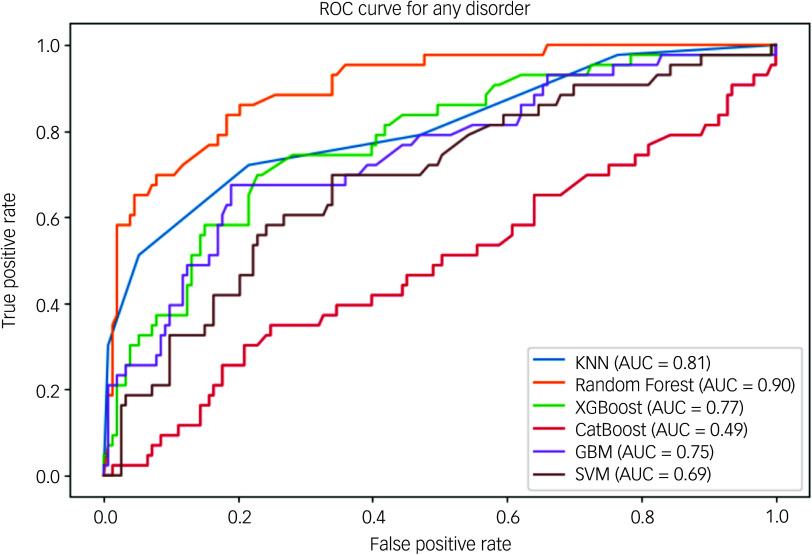
Area under the receiver operating characteristic curve of any mental illness. ROC, receiver operating characteristic; KNN, *k*-nearest neighbour; AUC, area under the curve; XGBoost, eXtreme Gradient Boosting; CatBoost, Categorical Boosting; GBM, gradient boosting machine; SVM, support vector machine.

Figure 2 shows the AUC-ROC curve of the algorithms for any disorder. The AUC-ROC is a widely used evaluation statistic in machine learning for binary classification models. The ability of a model to distinguish between positive and negative categories is a key performance indicator. The impressive AUC-ROC values of the models show how well they can distinguish between positive and negative categories. All methods, meanwhile, produced impressive AUC-ROC values. The best discriminatory power is obtained by the random forest AUC-ROC with a value of 0.90, the KNN AUC-ROC with a value of 0.81 and the XGBoost AUC-ROC with a value of 0.77. Claims of considerable discriminatory power are no longer supported by CatBoost’s AUC-ROC value of 0.49, and the previous findings of the SVM’s performance have been modified to reflect its new AUC-ROC value of 0.69. These adjustments guarantee correctness and consistency in how the metrics are interpreted. With the highest AUC-ROC value of 0.90, the random forest model outperformed the others in terms of producing accurate results among all models for any disorder and well-calibrated predictions. It can distinguish between positive and negative categories with competence and proficiency.

## Discussion

This study examined predictive factors for mental illness, defined as the presence of at least one condition among depression and anxiety using machine learning models. The best results are obtained by XGBoost in terms of F1 score of 71.01%, precision of 71.58% and competitive accuracy of 71.15%. The ability of XGBoost to generate trustworthy and confident predictions is further demonstrated by the lowest log-loss value of 0.56. The random forest followed with accuracies of 78.21% and AUC-ROC of 0.90. Feature importance analysis identified house type, age group and educational status as key predictors across models. CatBoost SHAP values highlighted the significant influence of these variables on the models’ predictions, with house type and age group consistently emerging as pivotal factors. Besides, the gain metric from the XGBoost model reinforced the importance of marital status and house type, with respective gains of 0.76 and 0.73, respectively.

Based on feature selection analysis and gain metrics, house type, marital status, occupation, family mental health history and monthly family income are significant predictors of mental illness. Previous studies have identified various predictors in different populations. For instance, Zhang et al^6^ found that for middle-aged participants, the top predictors included the ratio of family income to poverty, general health conditions and trouble sleeping, while adenosine triphosphate was the most important variable for males and it was general health conditions that were paramount for females. Among young employees, gender, physical health, job type and psychosocial factors are key predictors of depression.^9^ Tabares et al^10^ identified parental education level, alcohol consumption and social security affiliation as significant predictors of anxiety in young people. Another study found waist circumference, neck circumference, sleepiness, age, etc., to be critical predictors of severe obstructive sleep apnoea using SHAP plots.^22^ These findings highlight the critical role of sociodemographic and health-related factors in predicting mental health problems and provide valuable insights for targeted interventions and further research.

Interestingly, while factors such as marital status, occupation, mental health history and income did not show significant associations with mental illness in logistic regression analysis, housing quality emerged as a critical determinant. Our study found that individuals in government-provided housing were more likely to experience mental health problems compared to those in privately owned homes. This finding aligns with Pineda et al,^23^ who highlight the impact of socioeconomic factors, particularly housing quality, on brain health and mental health. Addressing housing disparities is therefore crucial for improving community mental health, and targeted policy interventions could play a vital role in mitigating these negative outcomes.

The comparative analysis of the SVM, random forest, KNN, CatBoost and GBM models revealed notable differences in performance metrics. XGBoost exhibited the highest accuracy of 71.15% and an AUC-ROC score of 0.77, indicating superior ability to distinguish between mental health conditions. This aligns with Tabares et al,^10^ who reported high accuracy using the random forest model for anxiety detection, and Chavanne et al,^11^ who highlighted the efficacy of ensemble models, including the SVM, in predicting anxiety. In contrast, the random forest model outperformed other models in terms of overall prediction power, accuracy, precision, log-loss and a high AUC-ROC value of 0.90. This is supported by Zhang et al,^6^ who demonstrated CatBoost’s effectiveness in predicting depression with high accuracy. The random forest model, while achieving lower accuracy (69.39%) and precision (64.36%), still demonstrated reasonable performance, consistent with Kim et al,^9^ where the random forest model showed strong performance in predicting depression. The relatively lower performance of the random forest model in our study might be attributed to the specific data-set and features used. AUC-ROC values revealed that all models performed well in distinguishing between mental health categories, but the SVM had the highest discriminatory power. This finding is in line with Chavanne et al,^11^ who reported the high efficacy of ensemble models, including the SVM, in anxiety prediction. On the other hand, the lower AUC-ROC values for the KNN and random forest models suggest potential limitations in their ability to differentiate between mental health conditions effectively, echoing mixed results from previous studies. For instance, Byeon^12^ found ensemble models combining SVM and random forest methods to be effective but did not specify their individual performance metrics, while another study^22^ demonstrated the GBM’s high AUC (0.857) in predicting sleep disorders. Collectively, these findings highlight the growing capacity of machine learning to enhance early diagnosis and treatment of mental health illness, emphasising the need for continued development and application in diverse populations.

One limitation of this study is its reliance on self-reported data, which may introduce bias or inaccuracies in reporting mental health conditions and sociodemographic factors. Besides, the cross-sectional design of this study limits the ability to infer causality. The study primarily aimed to explore associations rather than establish predictive or causal relationships. Future longitudinal studies are needed to confirm these findings and evaluate causality. While the machine learning models provide valuable insights, they may not fully capture the complexity of mental health, particularly the influence of unmeasured variables or interactions between predictors. It is important to note that the categorisation of house type may not fully reflect the living status of younger participants, who often reside with their parents, unlike elderly participants. This difference could affect the comparability of the housing variable across age groups. Finally, we acknowledge that no single area can perfectly represent the entire rural population of Bangladesh. Future studies could consider including multiple Upazilas to further enhance representativeness.

In conclusion, this study provides valuable insights into the risk factors for mental illness, specifically depression and anxiety, through the application of machine learning models. Key predictors identified include marital status, house type, family mental health history, and income, highlighting the significant role of sociodemographic variables in mental health outcomes. The analysis underscores the critical impact of housing quality, with individuals living in government-provided houses showing higher rates of anxiety, reflecting the broader influence of socioeconomic factors on mental health. Machine learning models, particularly XGBoost, demonstrated strong predictive performance, although the random forest model showed a superior AUC-ROC and discriminatory power. These findings emphasise the importance of considering sociodemographic and housing factors in mental health assessments and suggest that targeted interventions addressing these factors could enhance mental health outcomes. Future research should explore the causal relationships between these predictors and mental disorders and consider diverse populations to validate and extend these findings.

## Data Availability

The data is available upon reasonable request from the corresponding author.
